# A Singular Publication

**Published:** 1885-04

**Authors:** 


A Singular Publication.—We have recently received from a dentist practicing in South Carolina, a pamphlet with the following title: *The Dental Student*, March No. 1885, which appears to be published in one of the States north of the Ohio River. In this pamphlet, which is made up of extracts from other journals and of unconnected articles put together in the crudest manner, is one entitled "The Cummings Patent," which pretends to be the proceedings of a Committee appointed by a general meeting of the dentists of New York and Brooklyn held in 1876, to adopt measures to contest the Cummings Patent. From what we learn, this pamphlet has created the impression that steps are being taken at the present time to raise money for such a purpose, and inquiry has been made of the editor of The American Journal of Dental Science concerning this matter. Why such an article should appear at the present time is beyond our comprehension, as it relates to a matter long since disposed of. Perhaps the party whose name appears in this article as willing to receive at the time such a movement was made, as farback as 1876, the amounts to be subscribed, can throw some light on this singular emanation. We advise all who have received this pamphlet to pay no attention whatever to any apparent attempt to raise money for an object which no longer exists.

